# Detection of Different Pesticide Residues in Seasonal Leafy Vegetables of North Bengal Region, Bangladesh

**DOI:** 10.1002/puh2.70220

**Published:** 2026-04-28

**Authors:** Md. Mejbah Uddin Mithu, Sadia Akter Shormela, Md. Shahinul Islam, Mahfuza Mubarak

**Affiliations:** ^1^ Department of Public Health Daffodil International University Dhaka Bangladesh; ^2^ Wazed Miah Science Research Centre (WMSRC) Jahangirnagar University Dhaka Bangladesh; ^3^ Department of Public Health & Informatics Jahangirnagar University Dhaka Bangladesh; ^4^ Department of Public Health International University of Business Agriculture and Technology (IUBAT) Dhaka Bangladesh

**Keywords:** food safety, Fourier transform infrared (FTIR), pesticide residue, public health, vegetable

## Abstract

Food pesticide residue is a significant health issue affecting the population of developing nations, including Bangladesh. Nevertheless, there is minimal literature on the report of pesticide residues of commonly used pesticides in seasonal leafy vegetables in the North Bengal area. As such this study was meant to identify pesticide residues in raw leafy vegetables through Fourier transform infrared (FTIR) spectroscopy. Three frequently consumed leafy vegetables were collected and analyzed, including Indian spinach, spinach, and stem amaranth. Overall, four pesticides have been observed in the three types of vegetables (two insecticides and two fungicides) depending on the characteristic absorption peaks in the FTIR spectra. These compounds were thiamethoxam, carbendazim, mancozeb + metalaxyl, and dimethoate. These pesticide residues likely cause food safety and health‐related challenges to the population. To minimize the potential health risks, increased monitoring of pesticide residues in the vegetables and promotion of integrated pest management (IPM) approach with trainings the farmers on safe handling of pesticides are recommended.

## Graphical abstract

FTIR spectroscopy detected multiple pesticide residues—thiamethoxam, carbendazim, mancozeb + metalaxyl, and dimethoate—in commonly consumed leafy vegetables from North Bengal, Bangladesh. These findings highlight potential food safety risks and emphasize the need for regular monitoring and safer pesticide management practices.

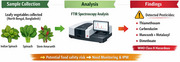

## 1 Introduction

Pesticide residues in food commodities are one of the major public health issues, especially in the developing countries, such as Bangladesh. Despite the fact that pesticides are very crucial in enhancing crop yield and food security, their inappropriate and overuse can result in build‐up of harmful residues in edible tissues of plants. Bangladesh is a nation that relies on agriculture, and in this case, pesticides are extensively applied in deterring pests and diseases among crops. BCPA [1] indicates that the pesticide industry makes a significant contribution to the national agricultural output. More than 60 vegetable species are now grown on about 470,414 h of land, and the growth rate of annual production of vegetables is 2.8%, which shows constant increase in recent years [2]. Although this can be beneficial in terms of national supply chain volume, as more pesticides are used, possibility of higher chances of contamination of fresh food with residues is also inevitable. One of the hazards of food safety that has appeared is pesticide contamination of vegetables [3].

Currently, about 84 pesticides are registered with 242 trade names in Bangladesh [4, 5]. Pesticides can be taken up by the plant leaves and roots and then translocate to the edible tissues [6], thus raising the chances of exposure through the human diet [7]. Some of the past studies indicated that vegetables that are contaminated with pesticides could be harmful to human health, especially when eaten raw or with minimal processing [8, 9, 10]. Past research undertaken in Bangladesh has reported pesticide residues in vegetables. Islam et al. [11] found pesticide residues in 27 of 42 vegetables samples (Narsingdi District), with 14 out of 27 having higher than the maximum residue limit (MRL). Likewise, Alam et al. [12] detected pesticide contamination in tomato and eggplant samples taken in Narayanganj District, and the levels of the remaining pesticides were above the MRLs in a number of instances. The aforementioned findings suggest that there is still a concern on the issue of pesticide residue contamination of vegetable supply chains [13].

Behavioral and psychosocial determinants usually affect the pesticide use and safety decisions of farmers. For instance, attitudes, knowledge, and perceived behavioral control (PBC) of farmers have a significant impact on the pesticide use behavior [14, 15]. Likewise, pesticide use and safety behavior is also determined by subjective norms, such as peer influence and social expectations [16]. Moreover, self‐efficacy and availability of safety materials influence adhering to safety precautions while handling pesticides in vegetable production zones [17]. Protective behavior has also been demonstrated to be dependent upon fear perception and response efficacy of farmers in developing countries [18]. Such drivers of behavior can lead to overuse or improper use of pesticides, which eventually leads to the likelihood of getting the residues in vegetables [19].

Though some studies have been conducted in Bangladesh to quantify pesticide residues through chromatographic methods, limited studies have been done regarding rapid screening of various pesticide residues in seasonal leafy vegetables through spectroscopic methods. Thus, the purpose of this study was to identify the pesticides residue in the regularly consumed leafy vegetables of the North Bengal area in Bangladesh through Fourier transform infrared (FTIR) spectroscopy. The results can help intensify the food safety surveillance and increase awareness about pesticide residue contamination in vegetables in Bangladesh and other regions with similar concerns.

The pesticides used in Table 1 are the commercial pesticide formulations that have been widely used and are readily available in the market according to the local agricultural suppliers and farmers in the study area. These pesticides were chosen as a reference standard of FTIR spectral comparison according to the patterns of the pesticide use that are reported in the North Bengal region in growing vegetables. Informal consultation was made with the local farmers and agrochemical retailers before the sample collection.

**TABLE 1:** Commercial pesticide formulations used as reference standards with respect to Fourier transform infrared (FTIR) spectral comparison.

Sl. no.	Trade name	Active ingredient	Formulation type	Company	Country
1	Tido	Imidacloprid (20 SL)	Liquid	Syngenta	Bangladesh
2	Actara	Thiamethoxam (25 WG)	Granule	Syngenta	Bangladesh
3	Knowin	Carbendazim (50 WP)	Powder	Syngenta	Bangladesh
4	Ridomil Gold	Mancozeb + metalaxyl (MZ 68 WG)	Granule	Syngenta	Bangladesh
5	Tafgor	Dimethoate (40 EC)	Liquid	Syngenta	Bangladesh

## 2 Materials and Methods

FTIR was chosen because the method is a fast, low cost, and nondestructive way of determining functional groups of organic compounds [20]. Compared to other chromatographic methods, including GC–MS/MS/LC–MS/MS that demand a complex preparation of samples and are also costly to operate, FTIR allows a quick qualitative screening of samples on the basis of their molecular vibration frequencies.

### 2.1 Study Area

**FIGURE 1:**
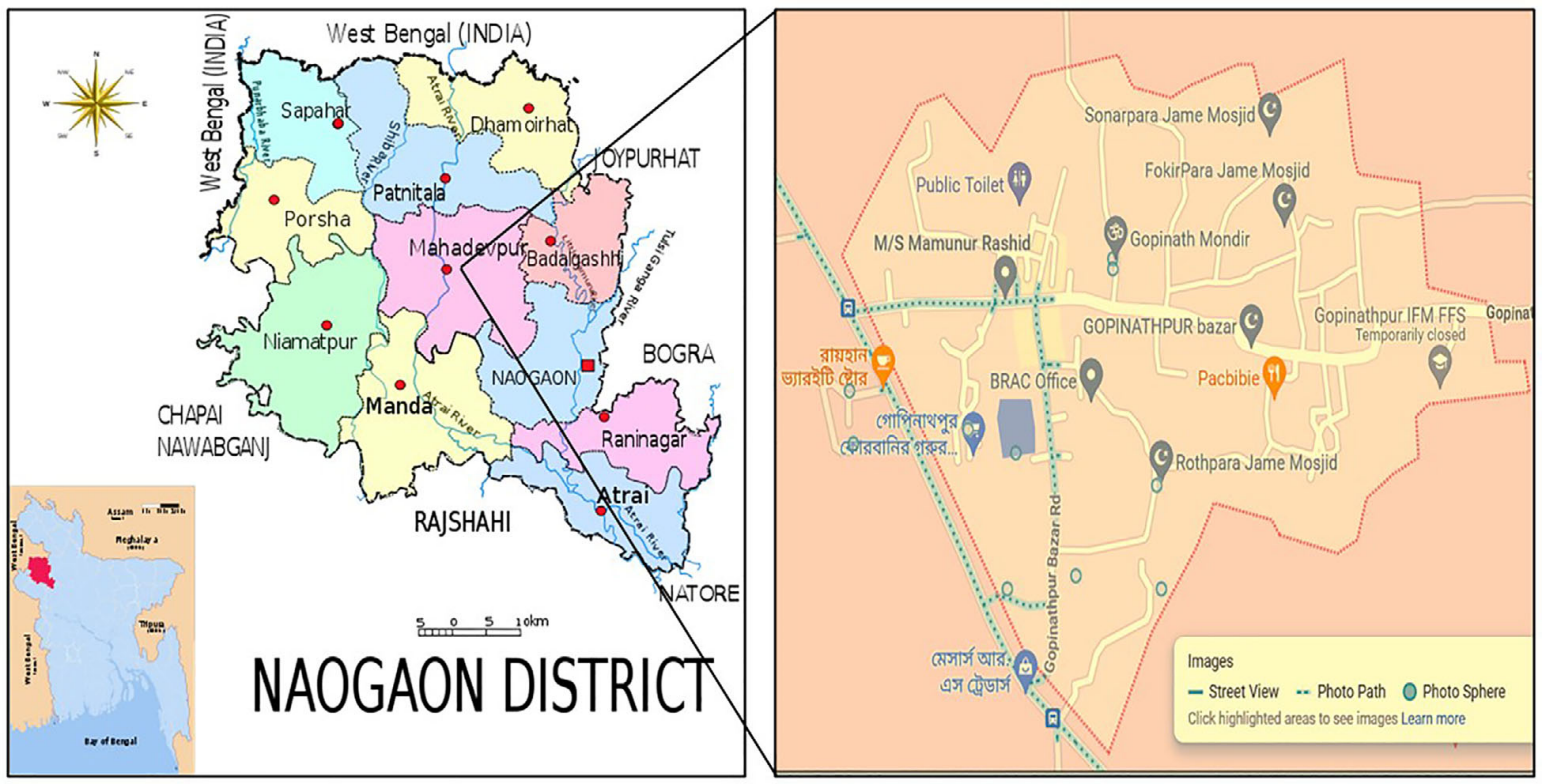
Study area.

The field research was carried out in the district of Naogaon, Bangladesh (in the North Bengal region, between 24°58′ N and 24°57′ N latitude and 89°05′ E and 89°06′ E longitude) Figure 1. The area belongs to the tropical wet and dry climate (Koeppen Geiger classification: Aw) with the clear monsoon season, hot summer temperatures, and cool winters [21]. The geology of the study region is mostly flood‐plain alluvial soil that is fertile and good in intensive agricultural production. Crops that are grown in this region are majorly rice, wheat, vegetables, mustard, and maize. The agricultural activities in the Naogaon District have been significantly commercialized, and the agricultural produce is sold to the local and regional markets and has been very important in generating income as well as the food supply of the households and the region.

This area will be a viable location to determine the possibilities of pesticide residues contamination of leafy vegetables with the high frequency of pesticides utilization in agriculture.

### 2.2 Sample Collection

Three leafy vegetables were chosen to analyze, including Indian spinach (*Basella alba*), spinach (*Spinacia oleracea*), and stem amaranth (*Amaranthus viridis*) (Table 2). The sampling was done in winter (December 2024 January 2025). Each of the vegetable types was collected randomly in large quantities of about 500 g (0.5 kg) in farmers who were growing crops in the chosen agricultural fields. All the samples were put down in dated and labeled polyethylene bags with the date of collection and location. Upon collection, the samples were taken to the Department of Public Health and Informatics, Jahangirnagar University, and stored at 4°C using a laboratory refrigerator before analysis. Samples were then transferred to the Wazed Miah Science Research Center to be analyzed by FTIR.

**TABLE 2:** Sample.

Common name	Scientific name	Local name
Indian spinach	*Basella alba*	Pui shak
Spinach	*Spinacia oleracea*	Palong shak
Stem amaranth	*Amaranthus viridis*	Data shak

### 2.3 Sample Preparation

Vegetable samples were dried in the oven at 100°C to a fine powder using a sterilized mortar and pestle. The dried vegetable powder was dried, and (the mixture) containing about 1 2 mg of potassium bromide (KBr) was pelleted. A hydraulic press was used to compress the mixture under pressure of 80 kN to create a transparent pellet that took 2 min. An FTIR spectrometer (the model: Prestige‐21, IR, Shimadzu, Japan) was then used to analyze the pellet with the spectral range of 4000–400 cm^−1^. Cleaning of all equipment applicable to the analysis was done with acetone to eliminate cross‐contamination. Before spectral acquisition, prepared pellets were put in desiccator so as to avoid absorption of moisture. Standard IR spectral assignments of C, H characteristic functional group frequencies were decoded depending on C, N, C, O, and C, H characteristic frequencies, respectively.

### 2.4 FTIR Analysis

The FTIR spectroscopy was used as a quick screening method to identify pesticide leftovers in vegetables. FTIR was chosen due to its low cost, fast, and nondestructive method of analysis with the potential to determine the presence of organic compounds depending on their functional group vibrations. Although the chromatographic processes like LC–MS/MS and GC–MS/MS can be regarded as the golden standards of the quantitative analyses of pesticide residues, they involve lengthy sample preparation, expensive operational rates, and instrumentation. Conversely, FTIR can be used to screen the qualitative analysis of pesticide‐related functional groups quickly, thus it is appropriate in the initial screening of pesticide residues, particularly in laboratory environments that have resource constraints. The principle of FTIR is to use an infrared radiation and direct it through a specimen and measure the absorbed energy at various wavelengths. The chemical bonds in the molecules vibrate at particular frequencies to give a characteristic absorption spectrum, which acts as a molecular fingerprint. Comparison of the obtained spectra with the reference spectra of the selected pesticide standards aimed at determining characteristic absorption peaks. Absorption bands identification was done with reference literature and standard infrared correlation charts [22, 23].

## 3 Results

FTIR spectroscopy was employed in identifying characteristic absorption peaks that have the potential of pesticide residues in three leafy vegetables, including Indian spinach (*B. alba*), spinach (*S. oleracea*), and stem amaranth (*A. viridis*). The spectra were matched with reference spectra of the chosen pesticide standards. They were identified with the help of the existing standard frequency reference tables of IR absorption [22, 23].

### 3.1 Indian Spinach

**FIGURE 2:**
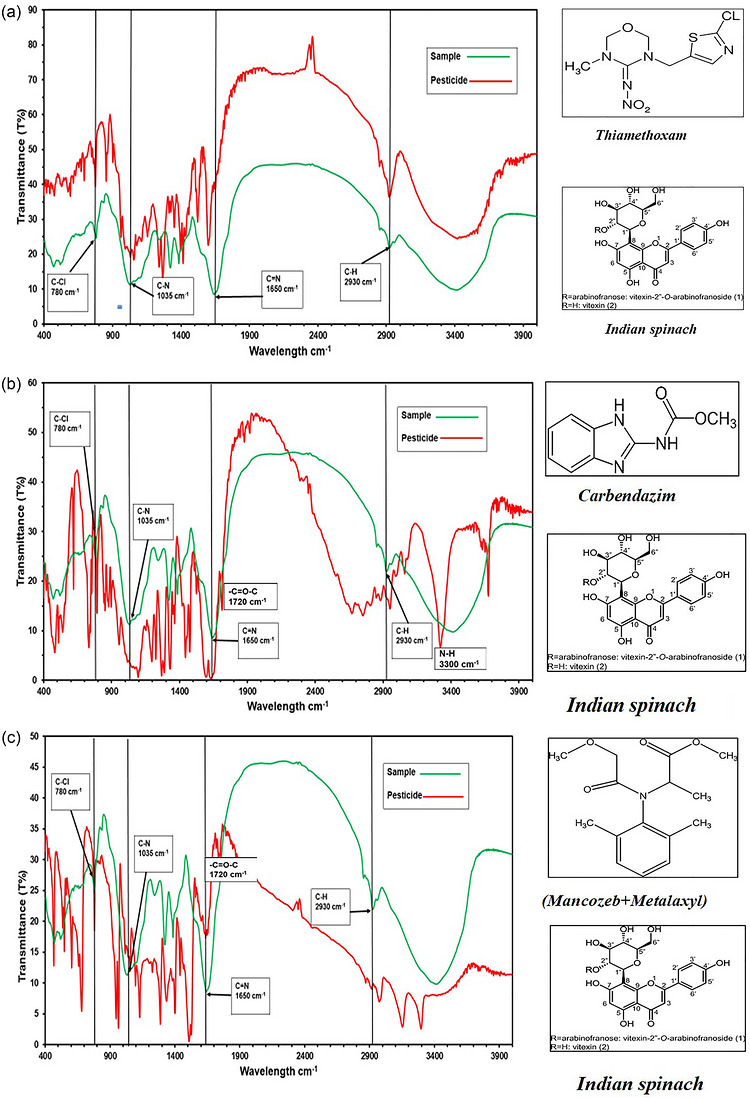
(a) Indian spinach versus thiamethoxam. (b) Indian spinach versus carbendazim. (c) Indian spinach versus mancozeb + metalaxyl.

FTIR spectrums of samples of Indian spinach displayed very strong absorption peaks at about 780, 1035, 1650, and 2930 cm^−1^. These absorption bands relate to functional groups, which are normally related to organophosphate and systemic fungicide compounds. Spectral comparison with pesticide reference spectra indicated that the spectrum was similar to thiamethoxam, carbendazim, and mancozeb + metalaxyl. The FTIR spectra of Indian spinach and corresponding pesticide standards are presented in Figure 2a,c.

### 3.2 Spinach

**FIGURE 3:**
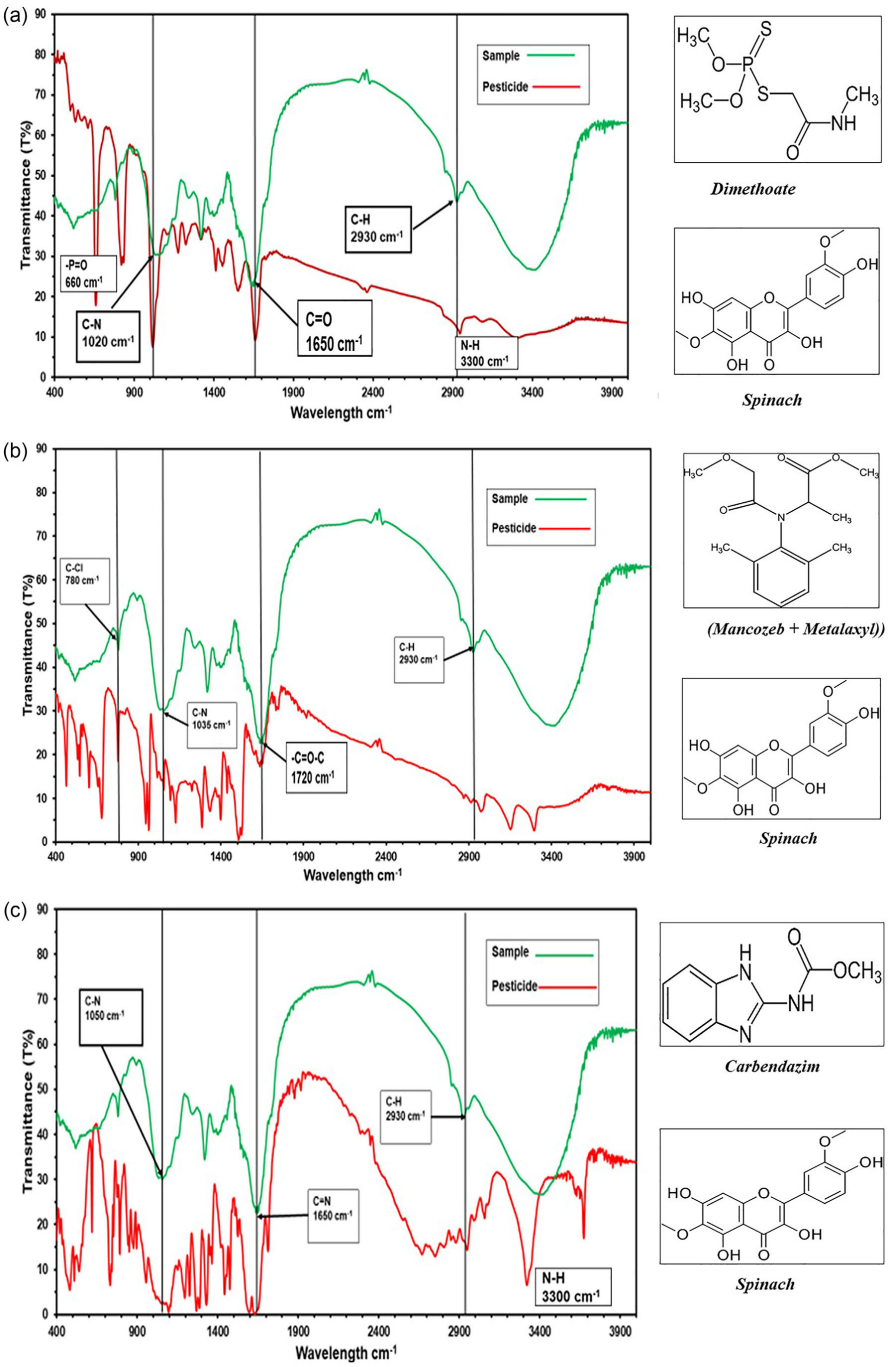
(a) Spinach versus dimethoate. (b): Spinach versus mancozeb + metalaxyl. (c) Spinach versus carbendazim.

The spinach samples as per the FTIR spectrum showed absorptions at around 780, 1020–1035, 1650, and 2930 cm^−1^. These peaks are in line with functional groups that are typical in organophosphate insecticides and systemic fungicides. Figure 3a,c presents the comparative FTIR spectra of spinach samples, showing notable similarities with the reference spectra of dimethoate, carbendazim, and mancozeb + metalaxyl.

### 3.3 Stem Amaranth

**FIGURE 4:**
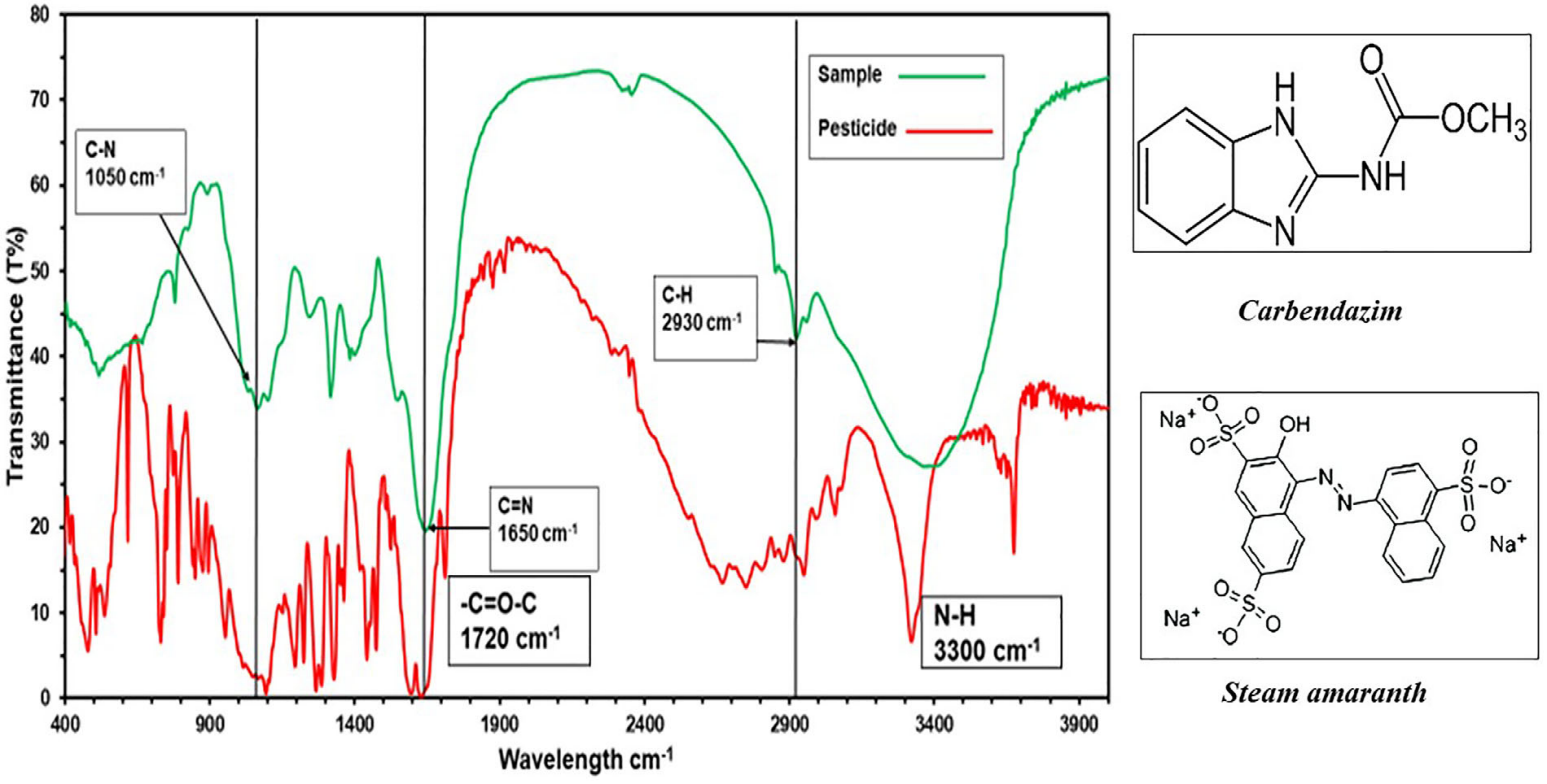
Stem amaranth versus carbendazim.

FTIR spectrum of the stem amaranth revealed the typical absorption peaks at around 1050, 1650, and 2930 cm^−1^. These bands are associated with functional groups that are seen in carbendazim reference spectra. Comparative analyses of spectral indicated the existence of carbendazim‐associated functional groups in amaranth samples of stems. The results of the FTIR spectrums are shown in Figure 4.

## 4 Discussion

This research found several residues of pesticides in three popular leafy vegetables that were grown in the North Bengal area of Bangladesh. According to the analysis on FTIR spectra, four compounds of pesticides were identified, which included thiamethoxam, carbendazim, mancozeb + metalaxyl, and dimethoate. Such compounds are in WHO Class II (moderately hazardous), which means that there are possible risks of public health in the case of recurring exposure to them in the diet. Previous studies have been done in Bangladesh to identify pesticide residues in vegetables that are commonly eaten. As an illustration, Islam et al. [11] identified diazinon, malathion, quinalphos, fenitrothion, cypermethrin, fenvalerate, and propiconazole in vegetables sampled in Narsingdi District, and some samples of them were over MRLs. Equally, Alam et al. [12] also found pesticide pollution in tomato and eggplant samples in Narayanganj District. Even though the main methods of the quantitative analysis in these studies were based on chromatography, the results are valid in proving that the issue of pesticide residue contamination is a persistent problem in Bangladesh. Similar results were also observed in other countries in South Asia.

Bhandari et al. [16] in Nepal emphasized the issue of unsafe practices in the use of pesticides by the farmers, and this is one of the reasons why the pesticide residue might be accumulated in food commodities. In India and Sri Lanka, multiple research studies have detected the traces of organophosphates and fungicides in leafy vegetables, which suggests that numerous issues related to the ineffective pesticide handling and the absence of postharvest intervals continue to poses a significant problem in the area. In Ethiopia, Chile, Mexico [24], Korea [25], and Egypt [26], pesticide residues in vegetables have been reported worldwide. These studies stated that several pesticide residues were found in individual samples of vegetables, and in some cases, even the recommended safety levels were surpassed. Even though the current research did not measure the concentrations of residues, the evidence that the typical functional groups were identified by FTIR analysis indicates that pesticidal substances are present in the edible plant tissues. The use of FTIR spectroscopy as a fast‐screening method of pesticide residues in leafy vegetables is one of the novel features of this research study. Although the use of more sophisticated methods like GC–MS/MS and LC–MS/MS can be regarded as gold standards of quantitative analysis of pesticide residues, the FTIR can be used as a means of preliminary detection of pesticides, which is cost‐effective, especially in environments with limited resources. This method can assist regular surveillance schemes in which use of expensive instrumentation is restricted.

It is alarming to note that leafy vegetables contain traces of organophosphate and systemic fungicides since they are known to cause neurotoxicity, endocrine disruption, and other chronic health complications with prolonged exposure. Because the consumption of leafy vegetables is usually not accompanied with extensive processing, the risk of dietary exposure can rise when the appropriate washing and handling methods are not implied.

All in all, this study has concurred with the evidence on a nationwide and regional scale showing continuous pesticide residues contamination in vegetables. Increasing the vigilance of the systems and encouraging safer practices of pesticides application are also needs to safeguard human health.

## 5 Conclusion

The study identified several pesticide residues in seasonal leafy vegetables in the North Bengal region of Bangladesh by FTIR spectroscopy. Altogether four pesticidal compounds, namely, thiamethoxam, carbendazim, mancozeb + metalaxyl, and dimethoate, were detected in the analyzed samples. These results show possible food safety issues and the necessity of frequent monitoring of the pesticide residue levels in vegetables. Specific training programs tailored to farmers on safe handling and use of pesticides are urgent. Moreover, there is a necessity to follow the government recommended dosage and preharvest intervals of the specific vegetables. In parallel promotion of integrated pest management (IPM) approaches focusing on low‐toxicity pesticides should be prioritized by the government to minimize public health risk.

## Author Contributions

**Md. Mejbah Uddin Mithu**: conceptualization, methodology, software, data curation, formal analysis, writing – original draft, writing – review and editing. **Sadia Akter Shormela**: conceptualization, data curation, writing – original draft, writing – review and editing. **Md. Shahinul Islam**: methodology, data curation, formal analysis. **Mahfuza Mubarak**: resources, supervision, project administration, writing – review and editing, visualization, validation, investigation.

## Funding

This research was fully self‐funded. All laboratory equipment and analysis costs were borne by the authors.

## Consent

This study did not involve human participants or personal data. The research was conducted using vegetable samples collected from farmers’ fields and analyzed in the laboratory using FTIR. Therefore, informed consent was not applicable for this study. All sample collection procedures complied with relevant institutional and ethical guidelines.

## Conflicts of Interest

The authors declare no conflicts of interest.

## Data Availability

The datasets generated and analyzed during the current study are available from the corresponding author upon reasonable request.

## References

[1] Bangladesh Crop Protection Association , Importance of Pesticide in Agriculture (Bangladesh Crop Protection Association, 2023), https://bcpabd.com/pesticide‐knowledge‐bank/importance/.

[2] A. K. M. Shahidullah , A. Islam , and M. Rahman , “Knowledge, Attitude, and Practice of Pesticide use by Vegetable Growers in Bangladesh: A Health Literacy Perspective in Relation to Non‐Communicable Diseases,” Frontiers in Sustainable Food Systems 7 (2023): 1199871, 10.3389/fsufs.2023.1199871.

[3] S. Begum , S. Sultana , M. Ahmed , and M. Azad , “Pesticide Residue Analysis From Winter Vegetables Collected From Six Markets of Rajshahi, Bangladesh,” Journal of Environmental Science and Natural Resources 12, no. 1–2 (2019): 43–50.

[4] M. Rahman and S. Khan , “Food Security in Bangladesh: Food Availability,” in National Workshop on Food Security in Bangladesh (Ministry of Food and Disaster Management, 2005).

[5] S. C. Barmon , B. M. Chaki , and Y. Wu , “Pesticide Use in Bangladesh: A Review on Potential Impacts,” Asian Journal of Environment & Ecology 16, no. 4 (2021): 224–241, 10.9734/ajee/2021/v16i430272.

[6] National Pesticide Information Center , Pesticides and Plants (National Pesticide Information Center, 2020), http://npic.orst.edu/envir/plantint.html.

[7] S. Akomea‐Frempong , I. W. Ofosu , E. D.‐G. Owusu‐Ansah , and G. Darko , “Health Risks Due to Consumption of Pesticides in Ready‐to‐Eat Vegetables (Salads) in Kumasi, Ghana,” International Journal of Food Contamination 4 (2017): 1–11, 10.1186/s40550-017-0051-1.

[8] A. Ahmed , M. Randhawa , M. Yusuf , and N. Khalid , “Effect of Processing on Pesticide Residues in Food Crops—A Review,” Journal of Agricultural Research 49 (2011): 379–390.

[9] B. Kumari , “Effects of Household Processing on Reduction of Pesticide Residues in Vegetables,” ARPN Journal of Agricultural and Biological Science 3, no. 4 (2008): 46–48.

[10] N. Thanki , P. Joshi , and H. Joshi , “Effect of Household Processing on Reduction of Pesticide Residues in Brinjal (*Solanum melongena*),” Advances in Applied Science Research 3 (2012): 2860–2865.

[11] M. Islam , K. Dastogeer , I. Hamim , M. Prodhan , and M. Ashrafuzzaman , “Detection and Quantification of Pesticide Residues in Selected Vegetables of Bangladesh,” Journal of Phytopathology and Pest Management 1, no. 1 (2014): 17–30.

[12] M. N. Alam , M. Z. Chowdhury , M. Hossain , M. M. Rahman , M. Rahman , and M. Khalil , “Detection of Residual Levels and Associated Health Risk of Seven Pesticides in Fresh Eggplant and Tomato Samples From Narayanganj District, Bangladesh,” Journal of Chemistry 2015 (2015): 1–7, 10.1155/2015/243574.

[13] G. Dinede , W. Bihon , L. Gazu , et al., “Assessment of Pesticide Residues in Vegetables Produced in Central and Eastern Ethiopia,” Frontiers in Sustainable Food Systems 7 (2023): 1143753, 10.3389/fsufs.2023.1143753.

[14] D. Khanal , U. Sapkota , G. Suwal , and P. Pandey , “Evaluation of Sustainable Biorational Pesticides for Managing *Helicoverpa armigera* on Tomato in Nepal,” Discover Agriculture 3, no. 1 (2025): 199, 10.1007/s44279-025-00308-2.

[15] U. Sapkota , G. Bhandari , M. Sapkota , et al., “Modeling Vegetable Farmers' Intention to Use Pesticides in Central Nepal: An Extended Version of the Theory of Planned Behavior,” Environmental Challenges 18 (2025): 101084, 10.1016/j.envc.2025.101084.

[16] G. Bhandari , A. Pandey , U. Sapkota , S. P. Singh , and H. Murano , “Pesticide Use and Safety Behavior Among Rice Farmers in Nepal: The Assessment of Theory of Planned Behavior and Potential Health Risk,” Environment, Development and Sustainability 27, no. 5 (2025): 1–30, 10.1007/s10668-025-06385-z.

[17] U. Sapkota , S. Adhikari , and G. Bhandari , “Farmers' behavioral Intention Towards Pesticide Safety in Nepal: An Assessment of the Extended Theory of Planned Behavior,” Journal of Environmental Management 389 (2025): 126185, 10.1016/j.jenvman.2025.126185. 40517631

[18] G. B. C. L. Bhandari , U. Sapkota , L. Fan , and V. Geissen , “Safety Behavior of Nepalese Strawberry Farmers as Reflected by the Protection Motivation Theory,” International Journal of Environmental Research 19, no. 3 (2025): 71, 10.1007/s41742-024-00726-y.

[19] F. M. Jallow , D. G. Awadh , M. S. Albaho , V. Y. Devi , and B. M. Thomas , “Pesticide Knowledge and Safety Practices Among Farm Workers in Kuwait,” International Journal of Environmental Research and Public Health 11 (2014): 3404–3419, 10.3390/ijerph110403404. PMC5409541 28338612

[20] M. Barnes , J. Sulé‐Suso , J. Millett , and P. Roach , “Fourier Transform Infrared Spectroscopy as a Non‐Destructive Method for Analysing Herbarium Specimens,” Biology Letters 19, no. 3 (2023): 20220546, 10.1098/rsbl.2022.0546. 36946131 PMC10031417

[21] M. C. Peel , B. L. Finlayson , and T. A. McMahon , “Updated World Map of the Köppen–Geiger Climate Classification,” Hydrology and Earth System Sciences 11 (2007): 1633–1644, 10.5194/hess-11-1633-2007.

[22] J. Coates , “Interpretation of Infrared Spectra, a Practical Approach,” in Encyclopedia of Analytical Chemistry, ed. R. A. Meyers (Wiley, 2000), 10815–10837, 10.1002/9780470027318.a5606.

[23] B. Stuart , Infrared Spectroscopy: Fundamentals and Applications (Wiley, 2004).

[24] R. Calderon , M. Gutierrez‐Hernandez , B. Sanchez‐Briceno , et al., “Assessment of Pesticide Residues in Vegetables Commonly Consumed in Chile and Mexico: Potential Impacts for Public Health,” Journal of Food Composition and Analysis 108 (2022): 104420, 10.1016/j.jfca.2022.104440.

[25] J. H. Park , S. H. Kim , H. J. Lee , et al., “Multi‐Residue Pesticide Analysis in Leafy Vegetables and Dietary Risk Assessment in Korea,” Food Control 135 (2022): 108783, 10.1016/j.foodcont.2021.108783.

[26] E.‐S. A. El‐Sheikh , M. M. Ramadan , A. E. El‐Sobki , et al., “Pesticide Residues in Vegetables and Fruits From Farmer Markets and Associated Dietary Risks,” Molecules (Basel, Switzerland) 27 (2022): 8072, 10.3390/molecules27228072. 36432173 PMC9695969

